# Improving Peer Relationships Through Positive Deviance Practices and the HOPE (Healthy Outcomes from Positive Experiences) Framework

**DOI:** 10.3390/ijerph22101550

**Published:** 2025-10-12

**Authors:** Laura Gallant, Catalina Borges, Alisha De Lorenzo, Curt Lindberg, Dina Burstein

**Affiliations:** 1 Tufts Medical Center, Boston, MA 02111, USA; dina.burstein@tuftsmedicine.org; 2 Department of Psychological and Brain Sciences, Main Campus, University of North Florida, Jacksonville, FL 32224, USA; c.borges@unf.edu; 3 Garden State Equality, Asbury Park, NJ 07712, USA; alisha@alishadelorenzo.com; 4 Complexity Partners, Waitsfield, VT 05673, USA; complexitypartners@gmail.com

**Keywords:** positive deviance, positive childhood experiences, peer relationships

## Abstract

Positive Childhood Experiences (PCEs), including supportive peer relationships, are crucial for optimal adult health and socioeconomic outcomes. As part of a broader initiative to address trauma in youth, we conducted a quality improvement project using a Positive Deviance (PD) approach. We aimed to improve peer relationships among members of the Asbury Park Boy & Girls Club and evaluate the feasibility of using a PD approach in a community-based setting. Using PD methodology, we identified practices used by staff to improve members’ experiences. Pre-intervention focus groups with staff and youth, discovery and action dialogues and staff observations identified positive deviants (PDs) and PD practices. PD practices were further defined during staff observations and developed into staff training. Post-intervention focus groups assessed perceived changes. Qualitative data was analyzed using deductive thematic analysis through the HOPE (Healthy Outcomes from Positive Experiences) framework domains of PCEs: Relationships, Environment, Engagement and Emotional Growth. In vivo coding generated subthemes, preserving participant language. Post-intervention focus group analysis suggested improvements in peer-to-peer relationships with club members referring to their peers as “nice” and “kind”, a contrast from pre-intervention findings. Findings were supported by club staff during member checking. These results suggest that the PD approach is a promising strategy for improving peer relationships and increasing access to PCEs in a community-based setting.

## 1. Introduction

The Secret Sits

We dance round in a ring and suppose,

But the Secret sits in the middle and knows. (Robert Frost)

### 1.1. The Positive Deviance Approach

The Positive Deviance (PD) approach, developed in the 1990s, is rooted in the idea that solutions to complex problems can often be found within the community itself. It was developed as a social and behavioral change process by Jerry and Monique Sternin who worked with communities in Vietnam to identify behaviors practiced by families whose children were well-nourished despite widespread malnutrition [1]. This methodology has since been applied across various fields, including public health, education, medicine, and organizational development, to uncover and amplify unconventional practices that lead to successful outcomes [2,3]. The PD approach emphasizes grassroots involvement and builds on existing strengths rather than imposing external solutions.

The PD method is specifically designed to address seemingly intractable problems in complex social settings. The PD approach is most effective when a concrete problem meets four key criteria. First, the issue must not be purely technical in nature; it should require behavioral or social change to achieve a solution. Second, PD is particularly suited to addressing persistent, “intractable” problems, especially in cases where conventional solutions have been unsuccessful. Third, the approach operates on the assumption that Positive Deviants (PDs) exist within the community; individuals or groups who, despite facing the same resource constraints, have already found ways to resolve the problem. Finally, successful implementation of the PD method necessitates sponsorship, prioritization, and a strong commitment to address the problem as a priority [3].

The PD approach was originally defined by four foundational steps: define the problem and establish measurable goals; identify individual groups or people, PDs, who achieve more favorable outcomes than their peers; discover the specific behaviors and strategies enabling their success (i.e., PD practices); and design opportunities for others to practice these behaviors. As the approach has been applied across diverse fields, it has evolved into a flexible methodology that often combines qualitative methods to generate hypotheses with quantitative methods to test them. Further refinements by organizations such as the Positive Deviance Collaborative describe five iterative steps: defining the problem and current practices; determining the presence of PDs; uncovering their uncommon yet effective behaviors; facilitating opportunities for others to adopt these practices; and systematically monitoring and evaluating progress [1].

### 1.2. Applications of PD Across Contexts

The PD approach has been successfully applied in various healthcare settings to enhance service quality and patient outcomes. A systematic review identified 27 studies utilizing PD in primary care, focusing on areas such as care effectiveness, chronic disease management, preventive care, prescribing practices, and health promotion [4]. In hospital environments, the PD approach has effectively reduced medication errors and hospitalizations. For example, a three-phase PD intervention program demonstrated a 0.12% decline in reported medication errors with each intervention phase [5]. Additionally, a PD approach was used to implement an antimicrobial stewardship program in outpatient dialysis centers as a way to reduce unnecessary antibiotic use and combat antibiotic resistance. Researchers were able to achieve a 6% month to month reduction in antibiotic dosing over the course of the 12-month study [6].

PD has also been effectively utilized in educational settings to enhance learning outcomes and student retention [7]. For example, a rural school district in Misiones, Argentina, used PD to address high primary school dropout rates. Workshops with teachers, parents and administrators identified local “positive deviant” schools with retention rates of 78–100% through Grade 3, compared to the district average of 56%. Many of the practices from the positive deviant schools such as fostering parent–school relationships, and adapting instruction to student abilities, resulted in improvements in retention and parent engagement [8]. Additionally, the UNICEF Office of Research—Innocenti initiated the Data Must Speak (DMS) Positive Deviance research program, which applies PD to schools in 15 countries. Using data to identify outperforming schools, DMS has uncovered grassroots solutions to educational challenges and collaborated with local partners to scale these solutions, thereby improving learning outcomes for children [9]. Similarly, the Stanford K12 Lab has explored the PD approach in K-12 education, engaging over 100 educators to identify positive deviant practices in areas such as student belonging and assessment equity, to then adapt and scale these practices across various classrooms [10].

While these initiatives demonstrate the impact of PD on youth populations in educational contexts, few studies have examined how the approach can be applied to foster positive childhood experiences (PCEs) and strengthen community-based support for youth. Therefore, the present study seeks to extend the PD literature beyond healthcare and formal education into the broader community context.

### 1.3. Positive Childhood Experiences

**Figure 1:**
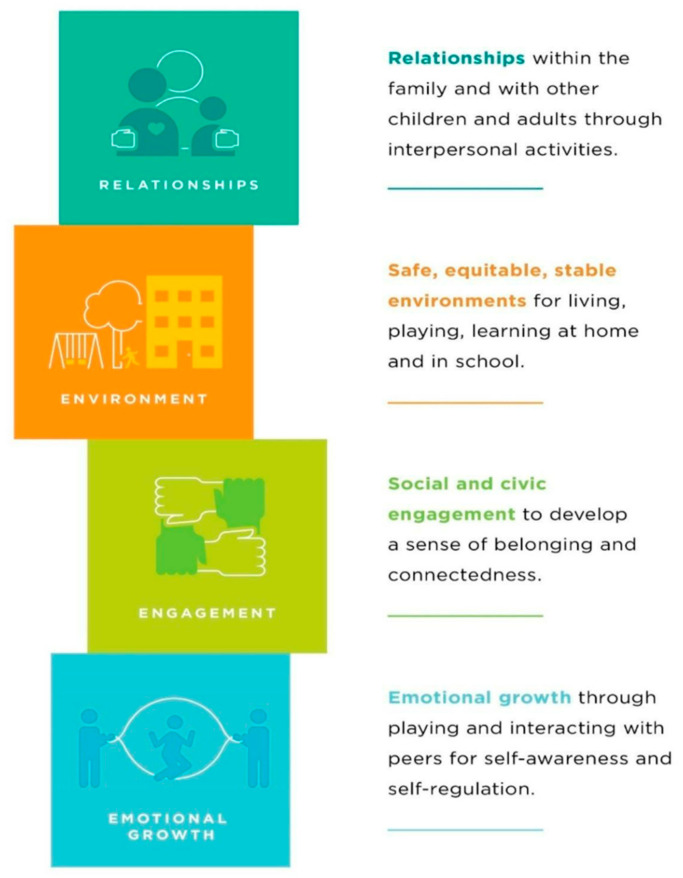
The four building blocks of the HOPE framework.

PCEs are crucial for fostering healthy development and mitigating the effects of adversity [11,12,13,14,15]. The HOPE (Healthy Outcomes from Positive Experiences) framework emphasizes the intentional promotion of PCEs, identifying four key building blocks essential for children’s well-being: nurturing, supportive relationships; safe, stable, and equitable environments; opportunities for constructive social engagement and connectedness; and the development of social and emotional competencies [16] (Figure 1). Research demonstrates that these positive experiences can counterbalance the negative impacts of adverse childhood experiences (ACEs), leading to improved health outcomes across the lifespan [12,13,14,17,18].

Youths’ relationships with caregivers, schoolteachers, peers, and others in the community can impact factors that promote PCEs, such as learning environments, perceptions of learning, and perceptions about themselves [19,20,21]. Given this, community organizations are recognized as essential for fostering youth well-being. Organizations like the Boys & Girls Club play a particularly important role in creating environments that offer opportunities for supportive peer relationships, mentorship, and meaningful engagement.

While the PD approach has been widely used to improve outcomes in healthcare and education, there is a paucity of research exploring how it can directly foster environments that promote PCEs in community-based settings.

### 1.4. Theortetical Framework

This study draws on several theoretical frameworks that explain how supportive peer relationships can promote access to PCEs. Resilience Theory empahsizes that protective factors such as strong peer relationships can help children overcome adversity and thrive [22,23]. Social Learning Theory further describes how peer interactions shape behaviors through modeling, observation and reinforcement [24]. When prosocial behaviors such as kindness and inclusion are modeled among peers, they become normative therefore strengthening emotional and social learning [25].

The PD approach aligns with these theories by identifying and amplifying successful behaviors within a community, those practiced by individuals who succeed in overcoming a problem despite access to the same resources [3]. This method utilizes a strengths-based approach similar to Positive Youth Development, which emphasizes the importance of building on youths’ assets [26]. The HOPE framework provides a structure to these approaches by organizing these key PCEs across the four building blocks of HOPE [16].

### 1.5. Boys & Girls Clubs of Monmouth County

The Asbury Park location of the Boys & Girls Clubs of Monmouth County (BGCM) serves as a vital resource for youth aged 5 to 18 in Asbury Park, New Jersey. Asbury Park, NJ, is a diverse community where 47.7% of the student population are Hispanic, 48.2% are Black, 3% are white, 0.1% are Asian, and 0.1% are American Indian or Alaska Native, and their graduation rate is 76%, below the state median [27]. BGCM staff prioritize fostering positive peer relationships among their members, recognizing the critical role these relationships play in youth development. Annual member surveys indicated that peer relationships were viewed as problematic. In the 2024 member survey, 61% of respondents indicated that peer relationships needed improvement.

This paper presents findings from one component of a broader initiative to address childhood trauma at BGCM, focusing specifically on examining whether a HOPE-guided PD approach can be used to improve peer relationships in this community-based youth-serving organization. Specifically, our research question was: Can a HOPE-guided PD approach be used to identify and amplify existing, effective practices that promote healthy and supportive peer relationships. Additionally, we evaluated the feasibility of applying the PD approach within this novel, community-based setting.

## 2. Materials and Methods

### 2.1. Setting/Participants

The project was conducted at the Asbury Park location of the BGCM in collaboration with Garden State Equality, an experienced PD facilitator (Lindberg), BCGM club leadership and the HOPE National Resource Center. Participants included club members and staff. Club members and staff participated in various aspects of the PD inquiry process including the discovery and action dialogues (DADs), focus groups, development and implementation of staff training and member checking. Project activities were conducted from 2023–2025. BGCM offers a safe and supportive environment for children and adolescents, serving 1850 youth [28]. Programs encompass homework assistance, technology centers, career exploration, and job assistance, aiming to empower young individuals to reach their full potential. The BGCM specifically focuses on serving children from challenging circumstances, providing them with essential resources and opportunities for personal growth. Through its comprehensive programs, the Asbury Park club plays a crucial role in fostering the development and well-being of the community’s youth.

### 2.2. Positive Deviance Inquiry

The PD inquiry was carried out through a combination of DADs, focus groups and staff observations. DADs engaged staff in identifying individuals who exhibited uncommon, successful strategies to foster positive peer relationships. Staff and members were prompted to share examples of staff who consistently handled challenges in unique and effective ways. PDs were identified through repeated mentions across the DADs and focus group sessions. For example, youth most often noticed staff for their distinctive practices, while staff highlighted peers they viewed as knowledgeable or skilled and turned to for advice and support. Practices were considered positive deviant practices if they were effective, feasible with existing resources, and not commonly employed by all staff. These insights were then explored further through targeted staff observations and focus groups to deepen understanding and assess how these practices could be adopted more broadly within the club environment.

#### 2.2.1. Procedure

Staff were informed about the project during regularly scheduled staff meetings. They were invited to participate in DADs and focus groups, with participation incentivized through the provision of lunch and gift cards. For staff observations, individuals identified by their peers were notified and asked for their consent to be observed in practice. Observations were scheduled in advance, and participants were informed of the specific dates and times these would occur. Verbal consent was obtained for all activities.

#### 2.2.2. Discovery and Action Dialogues

Discover and Action Dialogues (DADs), structured, participatory discussions, were conducted in five different sessions. Each DAD was facilitated by two members of the research team and included two or three BGCM staff. Each DAD lasted approximately 30 min. While the sessions were not audio- or video-recorded, detailed written notes were taken to document the discussions. A structured guide was used to facilitate these discussions. A total of 13 staff members participated in the DADs sessions, representing a range of roles including bus driver, mid-level staff, youth development professionals, and senior leadership. The DADs facilitation guide questions can be seen in Table A1 in Appendix A.

#### 2.2.3. Focus Groups

Pre-intervention focus groups were held with both staff and club members to support the PD inquiry process and gather baseline data. Four separate focus groups were conducted, two with staff and two with members. A total of 12 staff members and 12 club members, aged 8–14, participated in the pre-intervention focus groups. Club members reported attending the club for between one and five years. Semi-structured focus group guides were developed by the research team. Guides for both staff and member focus groups were informed by the study objectives and the HOPE framework. Guides included open-ended questions designed to elicit participants’ experiences at BGCM and perceptions of peer-peer relationships, peer-staff relationships, and staff practices. Three post-intervention focus groups were conducted, one with staff members and two with club members. These included one focus group with seven staff members and two focus groups with a total of 10 club members aged 8–14.

Club members were recruited via flyers sent home to parents or guardians via standard club emails inviting participation in the focus groups. Parents had the opportunity to opt-out for their child as per standard club procedure. Club staff were recruited via flyers distributed at the club. Verbal consent was obtained at the beginning of each focus group. Consent language was included at the beginning of the focus group guide. Staff were compensated with a $25 gift card. Members were treated to a pizza party as compensation for their participation. All focus groups were held at BGCM and lasted 45 min (members) to 60 min (staff).

Each focus group was staffed by two members of the research team, one acting as the facilitator and one as notetaker. All focus groups were audio recorded and later transcribed by a member of the research team.

#### 2.2.4. Staff Observations

Staff observations were conducted after the DADs and the pre-intervention round of focus groups. Structured observations were conducted on four staff members who were identified as PDs. Their roles included senior leadership, teen staff and youth development professional. The observer took notes as the identified PDs interacted with members throughout their day, observing how they each were executing their identified PD practices. Notes were taken detailing the observation of what is happening between the members and identified PDs as well as quotes from the interactions.

### 2.3. Intervention (Training) Design and Implementation

#### 2.3.1. Training Design

PD training was developed by the study team using learnings from the DADs, focus groups and direct staff observations. Training provided an overview of the practice, featured sample scenarios to demonstrate real-world application, included teach-back activities to reinforce learning, and offered opportunities for staff to ask questions and engage in interactive discussion. A detailed description of these training sessions can be seen in Table A2 in Appendix A.

#### 2.3.2. Training Implementation

Staff members identified as PDs during the focus groups and DADs were invited to lead the training segments that focused on their specific practice. They guided other staff members by explaining how they implemented these strategies in the daily work at BGCM and facilitated role-play activities to demonstrate the practices in action. The training was delivered twice, once at the all-staff summer camp training and again at the all-staff meeting held in the fall before the start of the school year. A total of 62 staff members participated in the training.

During the training, staff modeled scenarios based on identified PD practices. One scenario demonstrated how to support a member in balancing tasks such as homework, with other activities. Another focused on helping youth navigate challenges. A third scenario addressed conflict resolution and modeled how to de-escalate tension by creating a space for reflection. Another role play illustrated a strategy to engage club members reluctant to participate in activities by recognizing and amplifying their strengths.

### 2.4. Outcome Measures/Data Collection (Post-Intervention Focus Groups)

Our primary outcome measure was the quality of peer-to-peer relationships within the club setting. This was assessed through staff and member focus groups allowing for in-depth exploration of their perceptions and experiences.

Previously created focus group guides were updated to include specific inquiries about PD training and practices (staff) and peer-to-peer relationships (staff and members). Three separate focus groups were conducted, one with staff members and two with club members. All focus groups were held at BGCM. Recruitment strategies and compensation were identical to the initial round of focus groups. Post-intervention focus groups were audio recorded and transcribed.

### 2.5. Data Analysis

Following collection of data from pre- and post-intervention focus groups with staff and club members, qualitative thematic analyses were conducted. All sessions were audio-recorded and transcribed verbatim to ensure accuracy and facilitate analyses.

This study used a deductive thematic analysis approach that begins with predefined categories that guide the coding process [29]. The four building blocks of the HOPE framework (relationships, environment, engagement, emotional growth) [16] provided a structured lens for identifying and organizing key themes across the data. These domains served as parent codes for organizing the data. This approach allowed for structured analysis while allowing for flexibility in capturing emergent subthemes from participant responses.

Focus group transcripts were coded manually by two members of the research team. Within each HOPE domain, in vivo coding was used to develop subthemes that reflected the language and perspectives of participants. Coders independently reviewed and coded transcripts, then engaged in iterative discussions to compare interpretations, resolve discrepancies, and update the codebook.

Pre- and post-intervention transcripts were analyzed using the same framework to facilitate comparison. The team met to examine whether new codes emerged, if existing themes were no longer relevant, and if the language used by participants had changed following the intervention.

### 2.6. Member Checking

As a part of the qualitative analysis process, member checking was conducted with three staff members of BGCM to ensure the accuracy of the focus group findings. Member checking, also known as participant validation, is a process in qualitative research where findings are shared with study participants as a way to ensure accuracy and credibility of the reported results [30]. These staff members were presented with summaries of themes extracted from the focus groups and invited to share their reflections on the findings and how they aligned with their experiences. In addition, senior leadership obtained further input by consulting with other BGCM staff, allowing for broader, more diverse perspectives.

A summary of the project timeline can be seen in Table A3 in Appendix A.

## 3. Results

Results were analyzed throughout different phases of the project, outlined in Table 1.

### 3.1. Discovery and Action Dialogs (DADs)

The findings from the five DADs sessions with the Boys & Girls Club staff informed the development of the training by identifying key staff members and practices already in use. Staff described methods for supporting youth experiencing stress, fostering safe and supportive environments, reducing peer conflict, and promoting youth leadership.

Most staff reported using both structured and spontaneous emotional check-ins to support youth. One staff member explained, “Just like we do homework and say hi to them, we have emotional check-ins. We ask, ‘How are you doing today?’ That simple question makes a difference” Another shared, “Kids come there when they need a break or just someone to talk to.” Emotional check-ins were viewed not only as supportive for youth but also as important for deepening staff-youth relationships.

Several staff members identified strategies to reduce peer conflict by fostering collaboration and mixing peer groups intentionally. These approaches aimed to break down cliques and encourage cooperation. One staff participant described, “Getting members working on teams and mixing membership on teams helps. It builds new connections and keeps things from getting heated”. Staff also mentioned how they facilitated conflict resolution by guiding them to talk through disagreements and build empathy.

Staff responses also highlighted youth leadership and voice in programming. Specifically, participants mentioned the benefits of giving youth structured roles, such as being a “helper” or “assistant”. One staff member explained, “We need to provide leadership and helping opportunities for members… but it has to be within structure”.

### 3.2. Pre-Intervention Focus Groups

#### 3.2.1. Analysis of Staff Focus Groups

Relationships

Staff Support and Check-ins

Staff consistently emphasized the importance of building genuine relationships with members through active listening and talking to members throughout their day. Staff described practices that emotionally support youth such as “check-ins”, conversations, remembering children’s interests and making themselves available to members. As one staff member shared, “You just have to be patient with them, and eventually they come to you.”

Helping Members Connect

Staff responses reflected staff efforts to facilitate youth relationships through shared interests and intentional programming. Clubs and activities were structured to encourage collaboration and interaction, helping even the quieter children connect. For instance, one staff member remarked, “Even the quiet kids start opening up when they’re with people they have something in common with.”

Peer Conflict

Interpersonal conflict was a common concern, with staff noting that peer conflict often stemmed from emotional reactivity, personality clashes, or lack of empathy. One staff member explained, “Kids can be so mean to each other… Some have really intense personalities, and it changes the whole dynamic.” Responses also reflected staff strategies for preventing and managing conflict between members such as ensuring structure, activity planning and staff presence.

Environment

Supportive Environment

Staff felt well-supported by both their peers and leadership. They described a collaborative culture where they could rely on one another and seek guidance from supervisors. Specifically, they shared that they felt their ideas were welcomed, communication was easy, and leadership is “open” to feedback.

Safe Environment for Members

Although indoor settings were generally considered safe, some staff identified transitional times like bus rides as moments when safety could be compromised. Staff emphasized that youth generally feel safe at the club and have trusted adults they can turn to when needed. They referenced calming spaces like the “chill zone,” rule reinforcement, and adult intervention during transit (e.g., on the bus), as practices to promote safety for members.

Engagement

Members’ Voice

Staff described encouraging youth participation by asking for their input, tailoring programming to their interests, and finding ways to keep them involved. They emphasized choice, flexible programming, and voting on activities as engagement tools. Staff explained that this helps members feel ownership and excitement. One staff member explained, “I try to ask them at least a couple times a month what they want to do, and then they vote.”

Member Leadership

Staff described multiple practices where youth were given formal or informal leadership opportunities. These included Counselor-in-Training (CIT) roles, classroom job charts, leading attention-getters or even peer-led group management. Staff emphasized how these roles increased confidence, responsibility and a sense of ownership in the club. One shared, “Some of the older kids help out with the younger ones; they like feeling responsible.”

Emotional Growth

Managing Feelings

Staff described tools like journaling, “chill bags,” and structured programs like Smart Movers as instrumental in helping children identify and learn to express emotions. These strategies were praised for promoting self-awareness. For example, a staff member noted, “The chill bag… helps them calm down and name what they’re feeling.”

Talking Through Conflict

Staff emphasized approaches that involve talking through conflict rather than simply separating children. This includes mediating conversations between children, sitting in the “chill zone,” and using reflection strategies like “you say your side, then you say your side.” Staff noted that children often calm down once they feel heard and frequently resolve the issue themselves with support.

Themes and key quotes from the staff pre-intervention focus groups can be seen in Table 2.

#### 3.2.2. Analysis of Member Focus Groups

Relationships

Peer Conflict

Youth often described peer conflict including teasing, exclusion, rumors, and physical altercations, as a common occurrence, especially when emotions were heightened. Some members noted that conflict sometimes arises without provocation and contributes to feeling unsafe or isolated while at the club.

Supportive Staff

Members described positive relationships with staff. Children expressed that certain staff members were kind, approachable, and emotionally supportive, especially during moments of sadness, anger, or confusion. They emphasized how this support made them feel safe and cared for. For example, one shared, “She lets me sit there and calm down in a quiet place.”

Environment

Staff Helps with Conflict

Youth recognized that staff played a key role in conflict resolution. They noted that staff would often separate individuals, encouraging them to use calming strategies, or referring them to quiet spaces (i.e., “chill-zones”). A member noted, “If there are two people arguing, they’ll take one upstairs and one downstairs.”

Engagement

Member Leadership

Members described participating in leadership activities such as Torch Club, helping younger members, and organizing chores. This gave them a sense of pride and mattering. One stated, “Torch Club… they write down notes about what people think could be better about the club.”

Enjoyable Activities

Youth highlighted the wide range of activities like swimming, art, gym, clubs. Also described how these matched their interests and kept them engaged. Staff were credited with offering choice and making activities enjoyable. As one explained, “My favorite activity here is probably Color Wars.”

Emotional Growth

Staff Helps Manage Emotions

Youth reported that staff support their emotional needs by offering space, quiet zones, or the option to wait before discussing conflict. These strategies help youth regulate emotions safely and feel in control. One noted, “I’ll be in the office sometimes… they’ll send me to her office to calm down.”

### 3.3. Analysis of Staff Observations

After analyzing the data from the DADs and pre-intervention focus groups, a trained member of the research team conducted observations of four identified potential PD staff members. These observations confirmed which staff members were identified as PDs. The observations also further clarified PD practices and refined what these practices looked like with members.

### 3.4. Positive Deviants and Practices Identified

Analysis of pre-intervention focus group data, DADs findings and staff observations led to the identification of five PDs and three primary PD practice themes:

1. Emotional Check-ins: Emphasizing the importance of individual and group emotional check-ins to foster environments where youth feel heard, seen, and supported, ultimately contributing to effective problem-solving.
2. Peer-to-Peer Conflict Resolution: Promoting strategies for resolving conflicts among peers and encouraging collaboration through intentionally mixed team membership.
3. Youth Leadership and Voice: Creating structured opportunities for youth to take on leadership roles, contribute to program design, and reflect on their experiences within programs.

These three themes provided the foundation for staff training. Training included a description of each practice, sample scenarios to illustrate real-world application, teach-back sessions to reinforce understanding, and opportunities for staff to ask questions and engage in discussion. Additional themes and key quotes from the member pre-intervention focus groups can be found in Table 3.

### 3.5. Post-Intervention Focus Groups

#### 3.5.1. Staff Focus Groups

Relationships

Emotional Check-Ins

Emotional check-ins, both formal and informal, were a consistent part of daily routines. Staff used tools like feelings charts or simply asked “How are you feeling today?” regularly. One person shared, “We have them write it down now, and we talk about it—it really helps.”

More Staff-Member Connection

Staff noted improvements in their ability to connect with youth through consistency, emotional attunement, and shared experiences. They highlighted the importance of validating youth feelings and creating an emotionally safe atmosphere. Strategies included being present, listening closely, and checking in regularly. A staff member reflected, “I do enjoy connecting with these [kids] a lot and just being there for them.”

Members Using Tools

Staff reported perceived changes that members were utilizing emotion regulation tools. Staff shared that youth now use “cool off” strategies or self-initiated breaks more frequently. One staff member explained, “Now they’ll be like, ‘I need to cool off,’ and they walk away instead of getting mad.”

Peer Conflict

Staff reflected that despite improvements, persistent conflict remained among certain youth. Responses reflected a main theme that sometimes members “…just don’t get along”.

Environment

Member Comfortability

Staff reported that youth appeared more comfortable in the space and more likely to express themselves or ask for support. They described members as “more open” and “willing to come talk.” Staff attributed this shift to increased emotional safety and consistent routines. One staff member remarked, “They feel safe here. That’s why they come to us now.”

More Supportive Staff

Staff described becoming more approachable and emotionally responsive. Several noted a shift in how they respond to youth needs, like being more patient, present, and attentive. One staff stated, “They open up to me because I listen without judgment. That wasn’t always the case.” Staff consistently emphasized openness and modeling vulnerability as key strategies.

Stability & Structure

The introduction of new roles (e.g., “floor leaders”) and clearly defined staff responsibilities enhanced structure and predictability. Staff credited these changes with helping to manage transitions and reduce chaos. As one noted, “Now everyone knows what they’re supposed to do… it’s smoother.”

Engagement

Members’ Voice

Staff actively incorporated member feedback into programming decisions, increasing youth ownership. They described using tools like suggestion boxes or open discussions to gather ideas. A staff member explained, “They pick what activities they want… it’s their club, so their voice matters.”

Member Leadership

Staff observed more youth stepping into leadership roles, often through formal opportunities like Torch Club or informally during daily activities. These roles were associated with increased confidence and responsibility. “They help clean, lead the game… it’s beautiful to see them grow,” one staff member shared.

Emotional Growth

Managing Feelings

Staff reported greater use of calm, proactive strategies to manage youth dysregulation. They modeled emotion regulation and intervened early to prevent escalation. One staff noted, “I take a deep breath and show them how I calm down too.” This approach fostered a calmer group environment and reduced reactive discipline.

Staff Support & Strategies

Staff emphasized helping youth identify and use tools to regulate emotions independently. They described offering strategies like coloring, fidget items, or designated break spaces. “I told him, ‘Go take your time, then come back when you’re ready,’ and he did!” one staff recalled.

Members Help Each Other

Staff noted increased peer-to-peer support among youth, including checking in on each other or offering comfort. “She went over and gave her a squishy without being told…that’s new,” one staff member observed. These interactions were framed as signs of emotional maturity and empathy.

Members using Tools

A new theme emerged around youth independently requesting tools or support when upset. Staff celebrated these moments as signs of growth. “He came up and asked for the coloring book…he knew what he needed,” one said. This self-awareness was framed as a major success of the intervention. Additional post-intervention staff focus group themes and quotes can be seen in Table 4.

#### 3.5.2. Member Focus Groups

Relationships

Supportive Staff

Members consistently described staff as kind, emotionally available, and helpful during times of stress. Staff were named specifically for their ability to listen and offer comfort, with one youth sharing, “I like Mr. M because when I’m sad he makes me laugh.” Others explained how staff allowed them to take space or talk things through: “They’ll give you like chill stuff to like help you out, and if you wanna go outside and talk with them, they’ll go.”

Supportive Members

Youth also noted an improvement in peer relationships, including helping one another manage emotions or giving space when needed. One youth said, “Sometimes if someone’s mad, we give them like chill stuff or we just like talk.”

Peer Conflict

Although peer conflict was still acknowledged, members appeared to describe these moments with more emotional insight and understanding. One youth explained, “If someone’s mad they don’t always mean it…they just had a hard day.” While disputes still arose, members reported that staff support and peer patience helped manage these moments.

Environment

Staff Helps with Conflict

Youth described staff as actively involved in de-escalating arguments and preventing conflict from worsening. One youth shared, “When people start arguing, staff will just say, ‘Let’s go take a walk,’ or something like that.” These interventions appeared to be well received by members and helped maintain a calm environment.

Feeling Safer

Members emphasized feeling safe and cared for in the Club, largely due to consistent staff support and the predictability of routines. A member noted, “They make sure we’re okay… like if someone’s mad, they help them calm down.” This sense of security contributed to emotional openness and belonging.

Engagement

Enjoyable Activities

Members shared favorite Club activities, which included color wars, basketball, talent shows, and dance. These activities were framed as fun, exciting, and energizing. One participant shared, “I love color wars…it’s the best thing here. You get to do challenges with your team.”

Members’ Voices

Several members explained that staff had begun asking for their input when planning programming. One child said, “They let us pick sometimes… like if we wanna do a drawing contest or something.” This inclusion was associated with feeling valued and respected.

Member Leadership

Members discussed being given opportunities to lead or help run activities, especially older youth involved in programs like Torch Club. One member shared, “I help with chores and keep the little kids on track.” These moments gave youth a sense of responsibility and pride, with another noting, “I feel like a leader now.”

Emotional Growth

Staff Check-ins

Frequent and intentional emotional check-ins were highlighted. Members described staff asking “how are you feeling?” and using tools like emotion charts. One youth explained, “They ask us what color we are—like green if you’re good, red if you’re mad.” These check-ins made youth feel seen and supported.

Supportive Staff

Members described staff offering calm and accepting responses when youth became upset. “If I’m crying, they don’t get mad. They just let me sit and then come talk later,” one participant shared.

Staff Helps Manage Emotions

Members described moments when staff helped prevent or calm down emotional outbursts. “They let me walk away if I’m mad. Sometimes we go outside and talk,” one youth explained. These strategies helped youth feel in control and respected during hard moments.

Activities to Help with Feelings

Some youth described using squishy toys, breathing techniques, or taking time alone when upset. For example, one youth stated, “I just grab a squishy and breathe if I feel mad.” See Table 5 for additional quotes and themes from the members’ post-intervention focus groups.

### 3.6. Pre- & Post-Intervention Focus Group Comparison

When comparing the results from the first and second round of focus groups, we looked for new themes that emerged and themes that were discussed more frequently compared to the first round. Under the relationship theme, we did not see any new themes emerge; however, we did hear more specific examples of positive peer relationships. Emotional growth showed new themes of de-escalation, and member-initiated self-regulation, meaning that staff were helping create/allowing for a space for members to self-regulate. Utilizing a PD approach guided by the HOPE framework shows promise in promoting stronger peer relationships and enhancing the social–emotional environment in a youth-serving organization.

### 3.7. Analysis of Member Checking

The member checking activity revealed that utilizing a PD approach sparked meaningful change among both staff and club members. Staff reported greater emotional resilience, especially during high-stress moments with members, as well as a stronger, more supportive team dynamic built on mutual recognition and trust. One staff member stated, “The PD process helped me realize what I was good at as a staff member, and it was a different type of recognition.” Staff also noted that the PD initiative has helped empower members to take ownership of their behavior with another staff member stating, “our kids now see themselves as part of the solution, not just followers.”

Member checking also revealed the acceptability of using the PD approach in a community-based setting. One club staff member remarked, “What makes Positive Deviance such a good fit for the Boys & Girls Club of Monmouth County is that it doesn’t come in with all the answers, it helps us find the ones already working right here. There’s always that one kid, that one staff member, that one routine that’s making a difference. This approach helps us spot it, build on it, and spread it in a way that feels real and doable.”

## 4. Discussion

This quality improvement (QI) project demonstrated that utilizing a PD approach was likely effective in improving peer relationships among youth at the Asbury Park Boys & Girls club. This also represents the first known use of a PD approach to address peer relationships. Post-intervention focus group analysis suggested improvements in peer-to-peer relationships with club members referring to their peers as “nice” and “kind.” Members also talked about helping each other manage emotions and giving them space when needed. Staff noted a shift in peer relationships with members utilizing “cool off” strategies, from a PD practice promoting self-regulation, instead of engaging in a conflict. Positive reflections on peer relationships were notably absent in the pre-intervention focus groups but emerged in the post-intervention focus groups, as seen by the increased mention of the Supportive Members theme. These emergent themes align with social–emotional learning (SEL) competencies, such as understanding and managing emotions and maintaining positive relationships [31]. This suggests that the club community had existing SEL-related strengths that the PD approach helped surface and amplify. By engaging both staff and youth in identifying PDs and PD practices, and disseminating those practices via formal training, the PD approach fostered more supportive social environments and improved peer-to-peer relationships. Staff engagement throughout the project reinforced acceptability and sustainability.

In addition to the potential impact on peer relationships, the project offers promising evidence for the feasibility of using the PD approach in out of school, community-based environments. The adaptability of this method, the emphasis on engagement with the community and reliance on locally generated solutions, make it especially well-suited for organizations with limited resources.

Importantly, these findings align with broader efforts to promote access to key PCEs, which have been increasingly demonstrated to lead to improved health and socioeconomic outcomes [11,12,13,14,17,18]. The PD approach has potential to serve as a practical tool for fostering access to PCEs in numerous settings.

Supportive peer relationships not only contribute to a child’s sense of belonging and emotional security, they also help children build critical skills such as communication and conflict resolution [32,33]. The fact that club members described their peers as “nice” and “kind” in the post-intervention focus groups compared to the pre-intervention focus group where they shared “Sometimes they just be fighting for no reason” suggests a shift on how club members view each other. This demonstrates the potential utility of PD as a strategy to foster positive relationships and healthy development.

**Figure 2:**
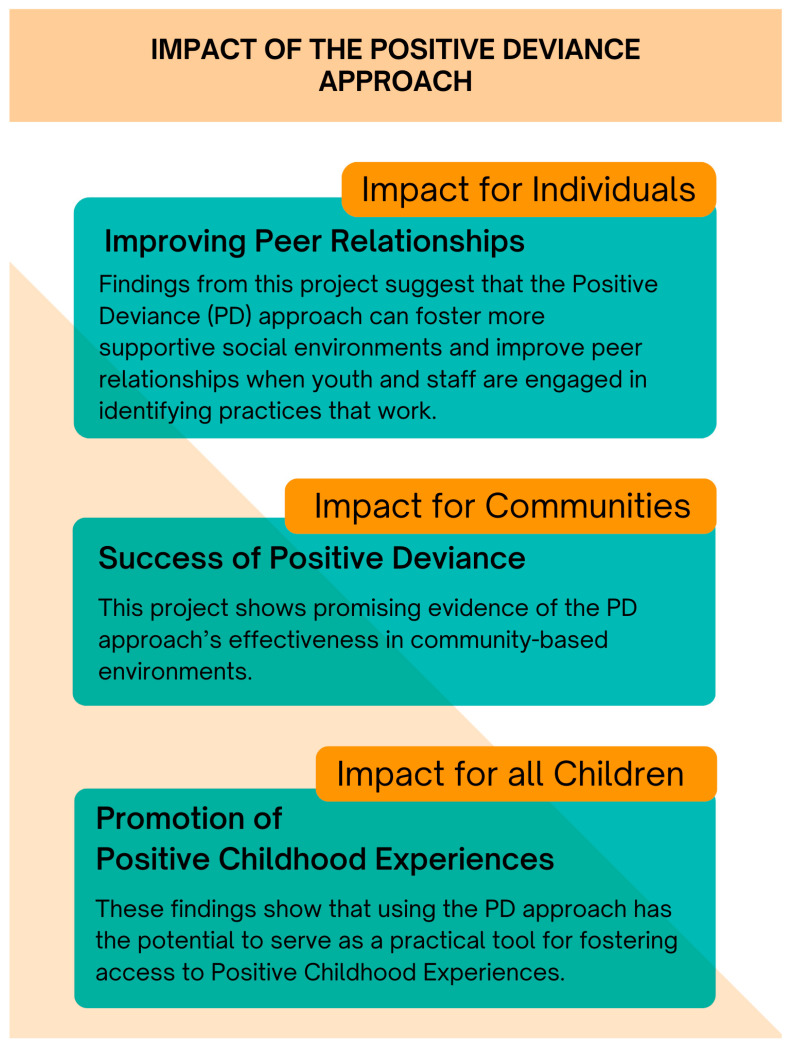
The levels of impact of the positive deviance approach.

This project also demonstrates the potential utility of using the PD approach in identifying and amplifying strengths within a community organization. By showing that peer relationships can be positively impacted through the identification and dissemination of PD practices, it offers a replicable model for other community-based organizations. This can be seen in the post-intervention focus groups from the increase in the More Staff-Member Connection theme, with staff members utilizing identified practices to create meaningful connections with members. These findings suggest that organizations aiming to improve youth social environments may benefit from using a PD approach. The PD approach has the potential to drive change at the individual, organizational, and community levels, contributing to expanded access to PCEs for all children (Figure 2).

This project demonstrates the effectiveness of using the HOPE framework to guide the PD approach in a community-based setting. The HOPE framework emphasizes that individuals and communities possess inherent strengths and abilities to promote PCEs. The PD approach builds on this, therefore driving large-scale change. By using the four building blocks of HOPE to guide the PD process we were able to identify PDs and their practices that increased the number of PCEs experienced by club members when implemented across BGMC.

While these findings are context specific, the PD approach is inherently adaptable and grounded in community engagement making it particularly suitable for replication by other organizations. Although the specific practices uncovered in this study may not be directly applicable to other organizations, the PD process as described could be replicated by a wide variety of community-based organizations seeking to achieve a variety of goals.

## 5. Limitations and Future Directions

While these results are encouraging, several limitations should be noted. This project was conducted in a single community organization therefore limiting generalizability beyond this setting. Findings may reflect contextual factors specific to the Asbury Park Boys & Girls Club such as staff culture, youth demographics and organizational policies and priorities. In addition, although the results are suggestive of improvements in peer relationships, the absence of a control group prevents us from attributing any observed changes solely to the PD intervention. While we shared in the introduction of the focus group guide that truthful answers are encouraged, we cannot be sure that youth and staff answered free from confirmation or acquiescence biases. Also, consensus for coding the focus group data was reached only through discussion among coders. Another limitation of this study is the lack of detailed demographic information for participants including race/ethnicity and socioeconomic status. Additionally, we did not collect data on staff years of experience with the club and prior work experience or familiarity with the PD approach. Finally, while the use of focus groups provided rich qualitative data and participant insights, the project did not include quantitative measures, as such the results are based on only qualitative data limiting the ability to draw definitive conclusions. Future studies should incorporate quantitative instruments and a more rigorous evaluation strategy to better assess the effectiveness of the PD approach in community-based settings.

## 6. Conclusions

This project demonstrates the promising potential of using a PD approach to enhance peer relationships among youth in a community-based setting. As the first known application of the PD model to address peer relationship, this work fills a critical gap in the field and contributes to the growing body of literature on trauma-informed and strengths-based interventions. By using a PD approach to surface and disseminate practices already working within the community, this approach empowered both youth and staff to take ownership of the problem and allow for positive change.

The work contributes to the field by offering a replicable, low-cost approach to addressing a problem, capitalizing on internal assets and solutions. For community organizations, especially those serving communities impacted by structural inequities, the findings offer a compelling case for utilizing a PD approach to promote positive outcomes.

## Figures and Tables

**Table 1:** Summary of results.

Data Source	Project Phase	Key Results
DADs * (Staff)	Pre-intervention	Emotional check-ins were a core practice Conflict resolution strategies identified Youth voice valued
Staff focus groups	Pre-intervention	Strong emphasis on relationship building Peer conflict identified as problematic Staff felt supported
Member focus groups	Pre-intervention	Peer conflict identified as problematic Staff seen as supportive Members felt they had opportunities for leadership roles and engagement
Staff observations	Pre-intervention	Confirmed positive deviants (PDs) Further clarified positive deviance (PD) practices
Staff focus group	Post-intervention	Improved peer relationships Acceptance of PD process and practices
Member focus groups	Post-intervention	Improved peer relationships Increased sense of agency
Member checking	Post-intervention	Acceptability of the PD approach Youth and staff felt empowered and saw themselves as part of the solution

* DADs = Discovery and Action Dialogues.

**Table 2:** Themes and Key Quotes—Staff Pre-Intervention Focus Groups.

Category	Themes	Quotes
Relationship	Staff Support and Check-ins Helping Members Connect Peer Conflict	“…emotional check ins that we do in the beginning… you can give them that space and just let them know that you’re there.” “Clubs help them build relationships…” “You just roll the balls out… that’s where you see the fights”
Environment	Supportive Environment Safe Environment for Members	“Communication is a big thing…leadership listens”
		2. “They feel safe to talk to us.”
Engagement	Members’ Voice Member Leadership	“I try to ask them at least a couple times a month what they want to do… and we make sure that they get to do that activity”
		2. “We have something called Torch Club… the kids run the program… they love that.”
Emotional Growth	Managing Feelings	“So sometimes to de-escalate something, we’ll be like… use the chill bag…”
	2. Talking Through Conflict	2. “…I feel like sometimes if you just take the child that’s like angry or mad and just walk with them and talk, you’ll learn a lot and they’ll tell you everything why they’re feeling like that. And nine times out of ten, you’ll hear it wasn’t even that situation that really triggered it. …”

**Table 3:** Themes and Key Quotes—Club Members Pre-Intervention Focus Groups.

Category	Themes	Quotes
Relationship	Peer Conflict Supportive Staff	“They say stuff to try and make you feel bad… like call you names or say you can’t sit with them.”
		2. “…I feel safe with the staff because they help me.”
Environment	Staff Helps with Conflict	“They separate us and let us calm down…they tell us, ‘Go to the chill zone and take some time.’”
Engagement	Member Leadership Enjoyable Activities	“They ask us to help with the little kids.”
		2. “I go to Dance Club and Keystone.”
Emotional Growth	Staff Helps Manage Emotions	“They give you time to calm down before talking to you.”

**Table 4:** Themes and Key Quotes—Staff Post-Intervention Focus Groups.

Category	Themes	Quotes
Relationship	Emotional Check-ins More Staff-Member Connection Members Using Tools Peer Conflict	“We ask how they’re doing, ask to see their gratitude card.” “I enjoy connecting with these [kids] a lot.” “I heard a kid say, ‘Take a deep breath’ to another one the other day.” “It’s just that some kids don’t like each other, and that’s not something we can fix with a tool.”
Environment	Member Comfortability More Supportive Staff Stability & Structure	“I want them to be comfortable… you’re not forced to do thing.” “I feel like it’s more okay now to ask for help.” “Structure really helped with transitions. It’s more stable now.”
Engagement	Members’ Voice Member Leadership	“After I finish an activity I’ll ask, ‘Did you like that? Do you want to do something different next week.” “They’ve been helping set expectations and rules.”
Emotional Growth	Managing Feelings Staff Support & Strategies Members Help Each Other Members Using Tools	“We try to get ahead of it now, before it turns into something.”
		2. “We made little chill kits… fidget stuff, coloring pages, stuff they can go to.”
		3. “She went over and gave her a squishy without being told…that’s new,”
		4. “They’ll say ‘hey man, breathe’, and it’s real sweet to see.”

**Table 5:** Themes and Key Quotes—Club Members Post-Intervention Focus Groups.

Category	Themes	Quotes
Relationship	Supportive Staff Supportive Members Peer Conflict	“They’ll look at me in my eyes and be like ‘Oh I got you’”
		2. “They’re nice and kind”
		3. “Because sometimes they argue and they bad and stuff.”
Environment	Staff Helps with Conflict Feeling Safer	“They actually stopped people from fighting and stuff.”
		2. “It feels safe now than before because they don’t let people scream anymore.”
Engagement	Enjoyable Activities Members’ Voices Member Leadership	“I like the new stuff we’re doing now; I like building things.”
		2. “They asked us what we wanted to do, and we picked slime.”
		3. “Sometimes we get to teach the rules to the little kids.”
Emotional Growth	Staff Check-ins Supportive Staff Staff Helps Manage Emotions Activities to Help with Feelings	“We do the mood meter sometimes.” “They give us hugs if someone is crying.” “If two kids are fighting, they talk to us to stop it.” “Sometimes I color when I’m mad.”

## Data Availability

Further inquiries can be directed to the corresponding author.

## Abbreviations

The following abbreviations are used in this manuscript:

PCEs	Positive childhood experiences
PD	Positive deviance
PDs	Positive deviants
BGCM	The Asbury Park location of the Boys & Girls Clubs of Monmouth County
HOPE	Healthy Outcomes from Positive Experiences
DADs	Discovery and Action Dialogues
DMS	Data Must Speak
CIT	Counselor in Training
QI	Quality Improvement

## Appendix A

**Table A1:** DADs * Facilitator Guide Questions.

Questions
How do you know or recognize when children are at risk or suffering from toxic stress or childhood trauma?
What do you help reduce the risk or help children heal from toxic stress or trauma?
What prevents you from taking these actions all the time (personal or organizational roadblocks?)
Is there anyone you know who is able to frequently reduce the risk, or overcome the barriers? Someone who despite the challenges has really caring and competent relationships with students, helps students regulate and gets better engagement? What do they do?
Do you have any recent experiences where engagement was exceptional? Less than ideal?
Do you have any ideas to help students who are experiencing toxic stress or childhood trauma?
What needs to be done to make it happen? Any volunteers?

* DADs = Discovery and Action Dialogues.

**Table A2:** Structure of Staff Training on Positive Deviant Practices.

Time	Workshop Content	Facilitator	Notes
5 min	Introduce PD * Work	BGCM ** senior leadership	
20–30 min	Run Scenarios (5 min each) Brief description (1–2 min) from each PD following their scenario describing their experience with using this practice.	Designated PD to present—Opportunities and Expectations (Youth Voice and Choice) Designated PD to present—Supportive Relationships (solving problems) Designated PD to present—Supportive Relationships (Conflict Resolution) Designated PD to present—Fun and Recognition Designated PD to present—Safe and Positive Environment (self-regulation)	5 key elements of positive youth development Opportunities and Expectations Supportive relationships Fun and Recognition Safe and positive environment Change the scenarios for the booster session
10 Minutes	Process Scenarios	What did you notice? Did you see an intervention that seemed to be effective? Which one?	
Lunch			
15 min	Teach Back Sessions	Break participants into 5 groups Assign each group to a PD PD researchers and BGCM staff leadership rotate around room to each small group PD’s review their role play scenario. Have participants volunteer to play a role in that scenario and role play the practices observed in the scenarios. Allow for several rounds of switching roles if time allows. 2 min before the session ends ask: What was it like? Can you imagine yourself using these practices in the club? End session and have group rotate to the next PD session.	
10 min	Facilitation idea: host circle conversation with the entire staff, use the questions to facilitate three rounds of conversation Other ideas?	How did it go? What did we learn ab these new practices? What did we notice about the impact? Do you have more ideas of how to reach more staff and members?	
Closing	Wrap up	BGCM senior leadership	

* PD = Positive Deviance ** BGCM = Boys and Girls Club Monmouth County.

**Table A3:** Project Timeline.

Project Month(s)	Project Phase	Activities
1–4	Planning	Assemble team Define goals Develop DADs * guide Develop focus group guides
5–10	PD ** Inquiry	Conduct pre-intervention focus groups Conduct DADs
11–15	PD Practice Identification and Training Development	Analyze PD inquiry data Identify PDs Staff observations Describe PD practices Translate PD practices into training
16–18	Implementation	Conduct PD practice training
21	Post-Implementation Data Collection	Conduct post-implementation focus groups
22–24	Data Analysis	Qualitative analysis of post-implementation focus group data
24	Member Checking	Member checking with club leadership and staff

* DADs = Discovery and Action Dialogues ** PD = Positive Deviance.
